# Cellular and Molecular Bases of Alcohol’s Teratogenic Effects

**Published:** 1994

**Authors:** Elias K. Michaelis, Mary L. Michaelis

**Affiliations:** Elias K. Michaelis, M.D., Ph.D., is chairman of the Department of Pharmacology and Toxicology and director of the Center for Neurobiology and Immunology Research, University of Kansas, Lawrence, Kansas. Mary L. Michaelis, Ph.D., is associate professor in the Department of Pharmacology and Toxicology and scientist at the Center for Neurobiology and Immunology Research, University of Kansas, Lawrence, Kansas

## Abstract

Research on the nutritional, hormonal, and cellular events regulating fetal development may help guide early interventions in children with FAS.

The condition known as fetal alcohol syndrome (FAS) was first described in the late 1960’s. FAS consists of a characteristic pattern of abnormalities resulting from exposure of the human fetus to alcohol during early development. These abnormalities include growth deficiency; a pattern of malformations affecting the head, face, heart, and urinary tract; and abnormalities within the brain that lead to various intellectual and behavioral problems in early childhood (Jones et al. 1974; Clarren et al. 1978; Shaywitz et al. 1980).

Many factors play a role in the development of FAS. Among these are the frequency and quantity of maternal alcohol consumption during pregnancy, the timing of alcohol intake during gestation, the stage of development of the fetus at the time of its exposure to alcohol, the nutritional status of the mother and her intake of other drugs, the genetic background of the mother and of the fetus, and the mother’s overall state of health. This article discusses the effects of alcohol on the cellular and molecular development of the fetus that may underlie the appearance of FAS in the newborn.

## Stages of Fetal Development

Human gestation is divided into two major periods: the embryonic period (up to 8 weeks of gestation) and the fetal period (from 8 weeks to delivery). It is during the embryonic period that malformations are readily produced by various drugs introduced directly into the maternal bloodstream or administered through the maternal diet. Chemical or physical agents that produce fetal malformations are called teratogens, from teraton, the Greek word for monster. Most teratogens show selectivities for certain organs, based on the timing of exposure of the embryo to the teratogen, the dose of the teratogen taken by the mother, and the sensitivity of the dividing cells of each primordial organ to the teratogen’s effects (Larsson 1973).

Organs and limbs of a developing embryo are formed from collections of specialized cells. Exposure of an embryo to a teratogen during the period when primordial cells of an organ are actively proliferating may have devastating effects on the formation of that organ.

Malformations of the head and face are the most common malformations in FAS and are correlated with exposure to alcohol during the embryonic stage and with the dose of alcohol consumed by the mother (Jones et al. 1974; Ernhart et al. 1987).

**Figure 1:**
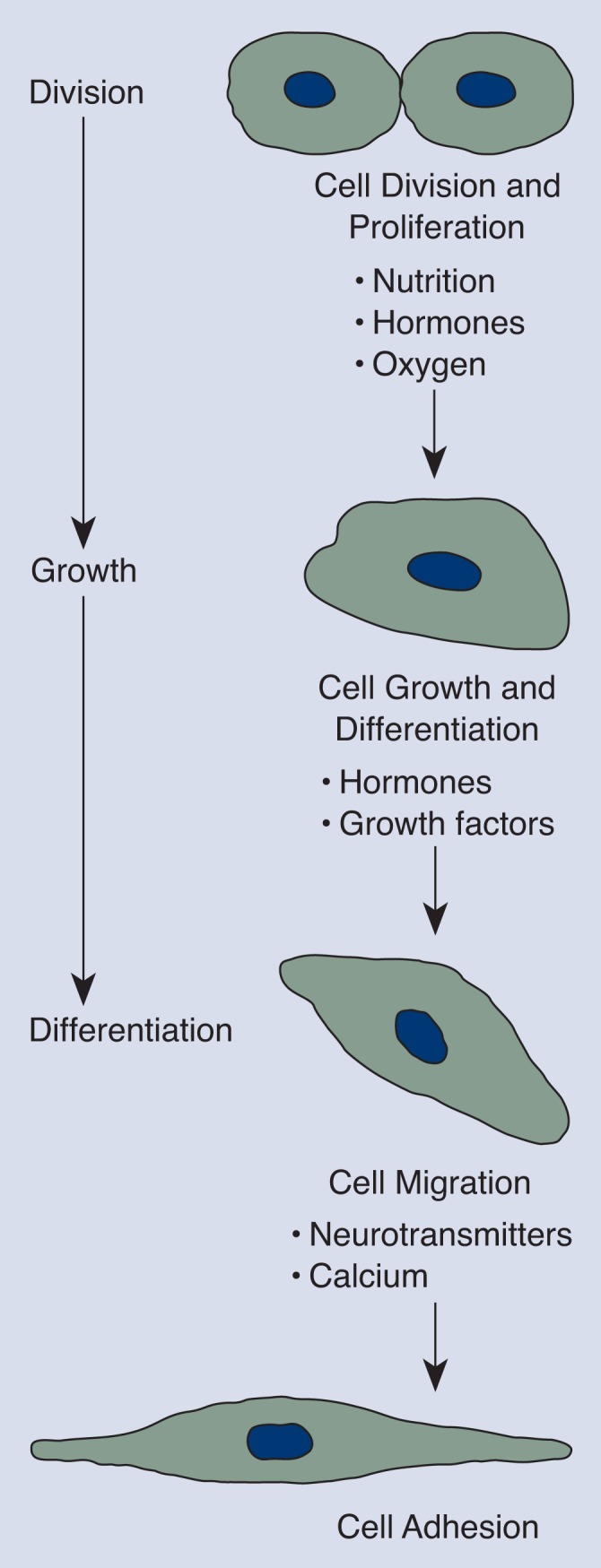
Cellular events that are potential targets of alcohol-induced disruption. Nutritional, hormonal, and cellular factors direct each stage of events.

Embryonic events at the cellular level that are potential targets of alcohol-induced disruption are summarized in figure 1. The first stage of events is cell division and proliferation. The second stage is cell growth and differentiation, by which cells become specialized in structure and function. For example, an embryonic nerve cell begins to develop the spines and branches that distinguish it, in part, from other types of cells. The final stage is the migration of maturing cells to their ultimate locations in the developing embryo, where they remain and adhere to the surrounding matrix of cells. Nutritional, hormonal, and cellular factors direct each of the above stages. Alcohol can affect many of these factors, thereby influencing organ formation and growth (figure 1).

## Mechanisms of Alcohol’s Embryonic Effects

Scientists have relied heavily on the use of experimental animals and in vitro models—whole embryos or cells grown in a test tube or petri dish—to help them determine the molecular and cellular events affected by exposure of embryonic tissues to alcohol. Such models are not ideal for studying how alcohol affects the human fetus, in part because other animal species do not metabolize alcohol exactly as humans do. However, such models enable scientists to study the effects of alcohol on fetal cells in the absence of confounding factors such as malnutrition, disease, or concomitant abuse of other drugs.

Although it has been suggested that the primary metabolic product of alcohol, acetaldehyde, could produce some damaging effects, all descriptions in this article of molecular and cellular mechanisms of alcohol-induced malformations are presented as direct effects of the alcohol on cells. The reasons for placing the focus on alcohol rather than acetaldehyde include the following:

- Alcohol is distributed rapidly and nearly equally in maternal and fetal tissues (Ho et al. 1972; Brien et al. 1983).
- Alcohol applied directly onto embryos in vitro under conditions in which no acetaldehyde is formed causes growth retardation (Snyder et al. 1992).
- Inhibition of the conversion of alcohol to acetaldehyde has no effect on the embryonic damage produced by alcohol (Blakley and Scott 1984).
- Measurable quantities of acetaldehyde are not present in the blood of alcoholics unless there is a genetic defect in the enzyme that breaks down acetaldehyde (Eriksson and Fukunaga 1993).

### Nutritional Factors

Children suffering from FAS commonly have low birth weight and remain small for their age (Jones et al. 1974). Normal growth and development during the gestational period requires the transfer of a constant supply of amino acids and glucose from the mother to the fetus across the placenta. Amino acids are the building blocks of proteins, the basic structural and functional elements of cells. The sugar glucose is the cell’s major fuel source, providing energy for the synthesis of proteins and the cell’s genetic material, or DNA.

Several studies with human placental tissue have shown that alcohol directly inhibits the transport of both amino acids and glucose (Snyder et al. 1986; Schenker et al. 1989). In experimental animals, the alcohol-exposed fetus suffers from selective amino acid deficiencies (Lin et al. 1990). Rat embryos exposed to alcohol in vitro exhibited marked growth retardation, decreased glucose metabolism, and diminished protein synthesis (Snyder et al. 1992). Thus, alcohol ingested by the mother deprives fetal tissues of the energy sources and materials needed for cell proliferation, growth, and differentiation.

In what may appear as a paradox, some organs of the developing fetus, such as the brain, are relatively protected during early periods of development against the damage caused by malnutrition, unless the malnutrition reaches very severe levels (Dobbing 1974). This points to the importance of the timing of exposure of fetal organs to alcohol; for example, Bonthius and West (1990) described the damaging effects of binge alcohol intake on different populations of nerve cells during later stages of development of the brain.

Supplemental glucose can partially reverse the developmental delays and metabolic abnormalities seen in embryos following alcohol exposure in vitro (Snyder et al. 1992). However, in experimental situations in which high protein diets were administered to rat mothers together with high amounts of alcohol, there was no diminution of fetal growth retardation associated with alcohol (Weinberg 1985). Therefore, nutritional supplementation may have only marginally beneficial effects on the fetus exposed to alcohol unless there are specific nutrients whose metabolism is blocked by alcohol.

Other nutritional deficiencies reported in either human alcoholic mothers or their infants include those of trace metals, such as zinc, and vitamins. As described in a review by Schenker and colleagues (1990), the presence of trace metal deficiencies during gestation in experimental models of FAS is controversial (Beer et al. 1992). With respect to vitamin deficiencies, there is evidence for decreased transfer of a form of vitamin B_6_ from an alcoholic mother to her fetus through the placenta (Schenker et al. 1992); this vitamin functions in protein metabolism. In addition, a possible defect in the metabolism of folic acid has been identified in some tissues of a fetus exposed to alcohol during gestation (Lin et al. 1992). Folic acid plays an important role in the synthesis of new DNA, and folic acid deficiencies during gestation produce malformations in the fetus. It is hypothesized that alcohol leads to a state of relative folic acid deficiency, which may account for some of the organ malformations seen in FAS.

There is no evidence of vitamin A deficiency in the liver or blood of fetuses from experimental animals fed alcohol through the gestational period. Instead, vitamin A appears to accumulate in the liver of the alcohol-exposed fetus (Leichter et al. 1991), suggesting that the vitamin is not being metabolized normally. This may be significant, because vitamin A is metabolized to a substance called retinoic acid. In many developing cells, retinoic acid functions as a chemical signal for the activation of DNA transcription, the first step in the synthesis of new proteins. Therefore, the lack of retinoic acid may be responsible for some of the developmental delays and malformations seen in FAS (Duester 1991; Pullarkat 1991).

### Hormonal Factors

The processes of cell proliferation and differentiation are controlled by many internal stimuli, both chemical and physical, emanating from the milieu of the developing tissues. The production and release of hormones from both maternal and fetal glands and from the placenta influence the formation and development of tissues as diverse as the brain and the palate. For example, in experimental animals exposed in the uterus to alcohol, there is a decrease in blood and brain concentrations of corticosteroid hormones during the newborn period (Kakihana et al. 1980; Taylor et al. 1982). These hormones regulate various aspects of metabolism and influence the organism’s response to stress. The deficiency in corticosteroid hormone production leads to deficits in the response of the newborn to stress. There are also deficits in the synthesis of a chemically related group of hormones, the sex steroid hormones. These hormone deficits may lead to the abnormal development of the brain, especially those specialized regions of the brain that differ between male and female and that control sex-related behavior (Rudeen 1992).

Thyroid hormone deficiencies during pregnancy also have been identified in the fetus born to human alcoholic mothers (Hernandez et al. 1992); these deficiencies may have a deleterious effect on the development of some tissues, in particular the brain (Hannigan and Bellisario 1990). In the cerebellum, a region of the brain that controls balance and posture, derangements in the maturation and migration of nerve cells to their appropriate locations caused by thyroid hormone deficiencies are similar to those observed when experimental animals are exposed to alcohol at or shortly after birth (Kornguth et al. 1979).

Recent advances in molecular biology have provided new insights into how apparently disparate influences such as nutritional and hormonal factors may act in a coordinated manner to produce growth retardation and malformations. Hormones and other chemical messengers (see below) exert their effects on cells by binding to specific receptor proteins on the cell surface or inside the cells.

It is now known that the receptors for steroid hormones, thyroid hormones, and retinoic acid are all members of a specific family of receptor proteins that regulate the transcription of DNA in a cell (Evans 1988). Under the influence of the appropriate hormones or other chemical messengers, these receptors can alter the rate of synthesis of proteins, thereby regulating the growth and differentiation of a cell. In addition, both thyroid hormone and retinoic acid regulate the synthesis of another hormone, growth hormone (Bedo et al. 1989). A relative reduction in growth hormone formation and release may contribute significantly to growth retardation in human FAS.

### Local Growth Factors

In addition to nutritional and hormonal factors, whose effects are widespread, various chemical substances regulate cell development locally in the region where they are produced. Some of these substances affect cell proliferation, growth, and differentiation, whereas others modulate cell migration and adhesion (figure 1). There is evidence that continued exposure of the fetus to alcohol may interfere with the function of at least some of these chemicals.

Among these chemical substances are a group of proteins known as growth factors. Growth factors acting through surface receptors on cells powerfully stimulate the transcription of the genetic material and the synthesis of many proteins. This leads to the growth of cells as well as to differentiation, such as the branching of nerve cells or development of contractile fibers in muscle cells.

Dow and Riopelle (1985) were the first to suggest that alcohol could diminish the effects of growth factors on nerve cells. Since then, studies have demonstrated reduced synthesis and release of a nerve growth-promoting factor in newborns exposed to alcohol during gestation (Heaton et al. 1992). Diminished protein synthesis by a newborn or fetus exposed in the uterus to alcohol has been repeatedly documented, especially in the brain (Tewari et al. 1992). Such diminution in protein synthesis may be the result of blunted action of growth factors in the fetus.

The brain regions of the hippocampus and cerebellum are particularly vulnerable to alcohol’s inhibitory effects on protein synthesis (Peters and Steele 1982). The hippocampus is a key brain area for human intellectual function and memory formation. Children with FAS have mild to severe deficits in these areas, with many of them exhibiting low intellectual functioning as determined by IQ tests, whereas others appear to have attention-deficit hyperactivity disorder^1^ (Shaywitz et al. 1980). Many of the children exhibit poor coordination of fine muscular movements. Some of these cognitive deficits and learning disabilities may be related to hippocampal damage produced during fetal alcohol exposure, whereas some of the movement disorders may result from damage to the cerebellum.

### Prostaglandins

Prostaglandins are local tissue chemicals derived from fatty substances, especially from arachidonic acid. Prostaglandin activity is markedly increased during exposure of the fetus to alcohol in the uterus (Schenker et al. 1990; Anton et al. 1990). These chemicals have powerful effects on the blood vessels of the uterus, placenta, and fetus. Their overproduction may be responsible for tissue hypoxia (lack of oxygen) brought about by prostaglandin-induced constriction of blood vessels.

Lack of oxygen functions as a trigger for cells in different tissues, such as the heart, to convert more arachidonic acid to prostaglandin (Kukreja and Hess 1992). This process would aggravate the preexisting hypoxia and, in the fetus exposed to alcohol, could lead to further tissue damage and growth retardation. This vicious cycle can be stopped by inhibiting the conversion of arachidonic acid to prostaglandin. Medications that can accomplish this include the anti-inflammatory drugs aspirin and indomethacin. These medications have been tested in experimentally induced FAS in animals and are of potential benefit for preventing some of the malformations caused by fetal alcohol exposure (Randall et al. 1987).

### Oxygen Effects

Diminished oxygen delivery to fetal tissues during maternal drinking may result either from the local release of prostaglandins as described above or from a direct action of alcohol on blood vessels of the placenta and umbilical cord. Alcohol has been shown to constrict these blood vessels (Mukherjee and Hodgen 1982), possibly by inducing the release of substances (endothelins) produced by cells lining the inner walls of the blood vessels (Tsuji et al. 1992). The increased production and release of these substances can markedly diminish blood circulation to tissues, inducing hypoxia and setting the stage for cessation or delays in cell proliferation, growth, or migration.

Following exposure of the fetus to alcohol during early gestation, the primordial cells that will form part of the nervous system and those that will form the tubules of the kidney show signs of damage or impending death (Gage and Sulik 1991; Petkov et al. 1992; Assadi and Zajac 1992). Some of these changes are similar to those observed in cells injured by hypoxia in adult animal tissues (Siesjo 1981). Hypoxia may, therefore, be the common mechanism that brings about developmental delays and malformations in several types of tissues.

Hypoxia also causes the formation of free radicals, toxic by-products of oxygen metabolism. Alcohol itself also induces free radical formation in some embryonic cells (Davis et al. 1990). Free radicals can damage the cell surface, allowing calcium to leak into and accumulate in the cells (Kukreja and Hess 1992). The abnormal accumulation of calcium in nerve cells may cause them to release neurotransmitters, chemicals by which nerve cells communicate. Some of these neurotransmitters may have toxic effects of their own on certain cells.

An organ that is particularly sensitive to hypoxia and that is most frequently damaged by alcohol abuse by the pregnant mother is the brain. The most common brain abnormalities in FAS are a decrease in the overall size of the brain and a diminution in the thickness of the outer layers of the brain (cortex). The decrease in size of the cortex is due to decreases in the total numbers of cells. Nerve cells depend on a continuous supply of oxygen and glucose to meet their energy needs. When nerve cells are stressed by decreased oxygen and glucose supplies, as may happen in FAS, they release large quantities of glutamic acid (Choi 1992). Glutamic acid is an amino acid that functions as a neurotransmitter. When this chemical is released from the branches of one nerve cell, it acts on the surface of another nerve cell, causing a transient electrical pulse. An excessive amount of this electrical activity can produce swelling and damage to nerve cells, primarily through entry into the cells of excessive sodium and calcium.

### Impaired Cell Migration and Adhesion

During brain development, certain types of cells are programmed to move to precise locations so that they can serve a specific role in the overall coordinated activity of the brain. In many FAS children, these nerve cells have failed to migrate to their appropriate sites. Scientists have reproduced these abnormalities in experimental animals and have referred to these clumps of misplaced cells as “brain warts” (Kotkoskie and Norton 1988; Miller 1993). It is assumed that these misplaced cells are not fulfilling their normal roles within the brain; therefore, this phenomenon may contribute to the observed mental deficits in FAS.

Very complex molecules in the local environment surrounding the maturing cells promote appropriate cell movement and adhesion. Alcohol apparently interferes with the cellular response to these molecules (Gatalica and Damjanov 1991). If the maturing cell does not detect the subtle gradients of these chemicals, then it will not initiate migration or will fail to complete it correctly. This is apparently what happens with various types of brain cells (Miller 1993).

The incorrect migration of some nerve cells within the brain also may be linked to the abnormalities in calcium regulation and the release of glutamic acid described earlier (Komuro and Rakic 1992, 1993). Gradients produced by small differences in local levels of glutamic acid or other neurotransmitters may be what orients these cells as they migrate toward their final destinations. If they are bombarded by excessive amounts of the neurotransmitter from all directions, as may occur during oxygen or glucose deprivation caused by maternal alcohol, these developing nerve cells may stop their migration and form a brain wart.

## Conclusions

Many crucial biochemical and cellular events are affected by exposure of the fetus to alcohol during gestation. It is too early to speculate whether any one of alcohol’s effects on molecular or cellular function is more significant than others. Considerable experimental effort will be required simply to validate that alcohol disrupts some of the same biochemical and cellular processes in humans as it does in experimental animals. Perhaps one day such information can be used to guide early interventions with FAS children—interventions that may help to reverse or minimize some of the deleterious effects of this widely consumed drug on the developing fetus.

## Footnotes

1 Attention-deficit hyperactivity disorder is a disorder of childhood characterized in general by levels of restlessness and distractibility that impair school and social functioning.
